# Popular Prejudices in Relation to Decay of the Teeth

**Published:** 1847-10

**Authors:** 


### *Popular Prejudices in relation to Decay of the Teeth.*

Every dental practitioner is almost daily compelled to listen to a long and tiresome story in relation to the cause of the disease he is about to treat. Perhaps in no department of Medical or Surgical Science you will find so many erroneous notions as in Dentistry. It is almost impossible where the mass of the community labor under false views in relation to any particular disease or mode of cure not to bring many under its influence. It is so much more easy to glide down with the current, unruffled by commotions around us, than to stem the tide of popular opinion and battle with ignorance sustained by prejudice, that many yield to that which they know to be false, rather than try to convince the skeptical. An oft repeated tale however unreasonable, to many minds, become as matter of fact, worthy of all credence. Some excuse there is for all this; for many, perhaps most capable, have but little time to argue disputed points with their patients, finding it more convenient and economical to stop his mouth with an instrument than with a logical argument.

Is it best, however, to let false views go uncorrected? While we think it unnecessary always to expose error when it will take more time than is convenient, yet it is perhaps our duty to very often give a *reason* for our *practice*, or press truth in contrast with error. Take, for example, the almost universal prejudice in relation to filing teeth, and if this is not combatted, you will find your patient holding you responsible for an operation where it is necessary to use the file to ensure success, and yet he objects. So in relation often to cleansing the Teeth. They have their teeth coated with tartar, irritating the gums and loosening the teeth—yet they fear its removal with an instrument will destroy the enamel of the teeth. As well might you expect to cure an inflamed eye, caused by the lodgment therein of a foreign body without its removal as relieve your patient without the removal of the tartar. Yet we have known reputable, scientific and distinguished practitioners of medicine hold these same views. Yes, and we have known *Dentists*, yes, obsequious Dentists, succumb to all this from such to secure their *vast* influence. We purpose, however, taking up some of these erroneous notions successively, and treat them as candidly as we can, so that the readers of the Register may determine how far they are destructive to the dental organs.

In the next number we will try and investigate the effects of Mercury on the Teeth, and see if it really occasions such terrible havoc in the Dental apparatus, by causing decay as it is said to. Thereafter we shall take up the subject of filing, cleaning, and various other popular notions in relation to the cause of decay of the Teeth.—Cin. Ed.

